# Management of Hypertensive Emergency in the Setting of Primary Aldosteronism Complicated by Heart Failure With Reduced Ejection Fraction

**DOI:** 10.7759/cureus.47545

**Published:** 2023-10-23

**Authors:** Tauseef Sarguroh, Aliziya Punjwani

**Affiliations:** 1 Internal Medicine/Nephrology, Franciscan Health, Olympia Fields, USA; 2 Internal Medicine, Midwestern University Chicago College of Osteopathic Medicine, Downers Grove, USA

**Keywords:** dilated cardiomyopathy (dcm), guideline-directed medical therapy (gdmt), combined systolic-diastolic dysfunction, the rales study, aldosterone-renin ratio, mineralocorticoid receptor antagonist, hfref: heart failure with reduced ejection fraction, primary aldosteronism, hypertensive emergency, spironolactone

## Abstract

We present a case of a 49-year-old man with a past medical history of uncontrolled hypertension and alcohol use disorder presently in sustained remission who presented to the ED with shortness of breath. He was admitted for the management of hypertensive emergency and hypokalemia and was later found to have primary aldosteronism complicated by heart failure with reduced ejection fraction. The patient’s treatment-resistant hypertension as well as hypokalemia, which was refractory to repletion, resolved with mineralocorticoid-receptor-antagonist pharmacotherapy. After a single oral dose of spironolactone 25 mg, the patient's mean arterial pressure decreased by approximately 26.5%. Spironolactone 25 mg was continued twice daily not only as the mainstay treatment for primary aldosteronism but also to optimize guideline-directed medical therapy for the treatment of heart failure with reduced ejection fraction.

## Introduction

Primary aldosteronism (PA) classically manifests with hypertension (HTN) that is resistant to three classes of antihypertensives, one of which is a diuretic, as well as metabolic alkalosis [1]. Although hypokalemia is an important characteristic of PA, less than 37% of patients with PA present with it [2]. A positive screen for PA consists of an elevated plasma aldosterone concentration (PAC) that is greater than 15 ng/dL and a decreased plasma renin activity (PRA) such that the aldosterone-renin ratio (ARR), which equals PAC/PRA, is greater than 25 ng/dL. According to the 2016 Endocrine Society guidelines, in subjects with spontaneous hypokalemia, a PAC >20 ng/dL and a PRA <1.0 ng/mL/hr, confirmatory testing (e.g. IV normal saline infusion test, oral salt suppression test, captopril challenge test) is not necessary to establish a diagnosis of PA [3]. Once PA is diagnosed, cross-sectional imaging is obtained to check for the presence of an aldosterone-producing adrenocortical adenocarcinoma, which would necessitate an adrenalectomy [4]. If there is no evidence of an adrenocortical adenocarcinoma on cross-sectional imaging, willing surgical candidates should undergo adrenal vein sampling (AVS) to differentiate between a unilateral and a bilateral disease process. Unilateral aldosterone overproduction, most commonly resulting from an aldosterone-producing adenoma and less commonly secondary to primary adrenal hyperplasia, is treated with an adrenalectomy. Contrastingly, idiopathic bilateral adrenal hyperplasia, which causes aldosterone overproduction bilaterally, is treated with a mineralocorticoid receptor antagonist (MRA) [4,5].

Potential long-term complications of untreated PA, such as cardiac hypertrophy and fibrosis as well as vascular smooth muscle hypertrophy, result from a chronically hypertensive and hypervolemic state secondary to the direct effects of excess circulating aldosterone (and hence, unregulated sodium reabsorption) in the cardiovascular system [6]. In PA, excess aldosterone enables the effects of cardiotrophin-1 which in turn leads to left ventricular (LV) hypertrophy [7]. Aldosterone potentiates reactive oxygen species, intercellular adhesion molecules, macrophages, and interleukin-6, promoting a chronic inflammatory state. Aldosterone also contributes to LV fibrosis by enhancing the function of fibroblasts, matrix metalloproteinase, and tissue inhibitor of metalloproteinase-1. LV remodeling, which is characterized by LV hypertrophy and fibrosis, leads to LV systolic and diastolic dysfunction [7].

## Case presentation

A 49-year-old man with a past medical history (PMHx) of poorly-controlled HTN and noncompliance with prescription antihypertensive drug therapy as well as alcohol use disorder (six beers daily for 15 years), in sustained remission, presented to the ED with complaints of worsening shortness of breath since testing positive for severe acute respiratory syndrome coronavirus 2 (SARS‑CoV‑2) two months prior as well as a 10-day history of bilateral lower extremity edema. He denied any associated fevers, chills, chest pain, palpitations, orthopnea, paroxysmal nocturnal dyspnea, headache, dizziness, pre-syncope, nausea, history of illicit drug use, recent exposure to sick contacts, recent foreign travel and/or any known PMHx or family history of coronary artery disease or heart failure (HF).

**Figure 1:**
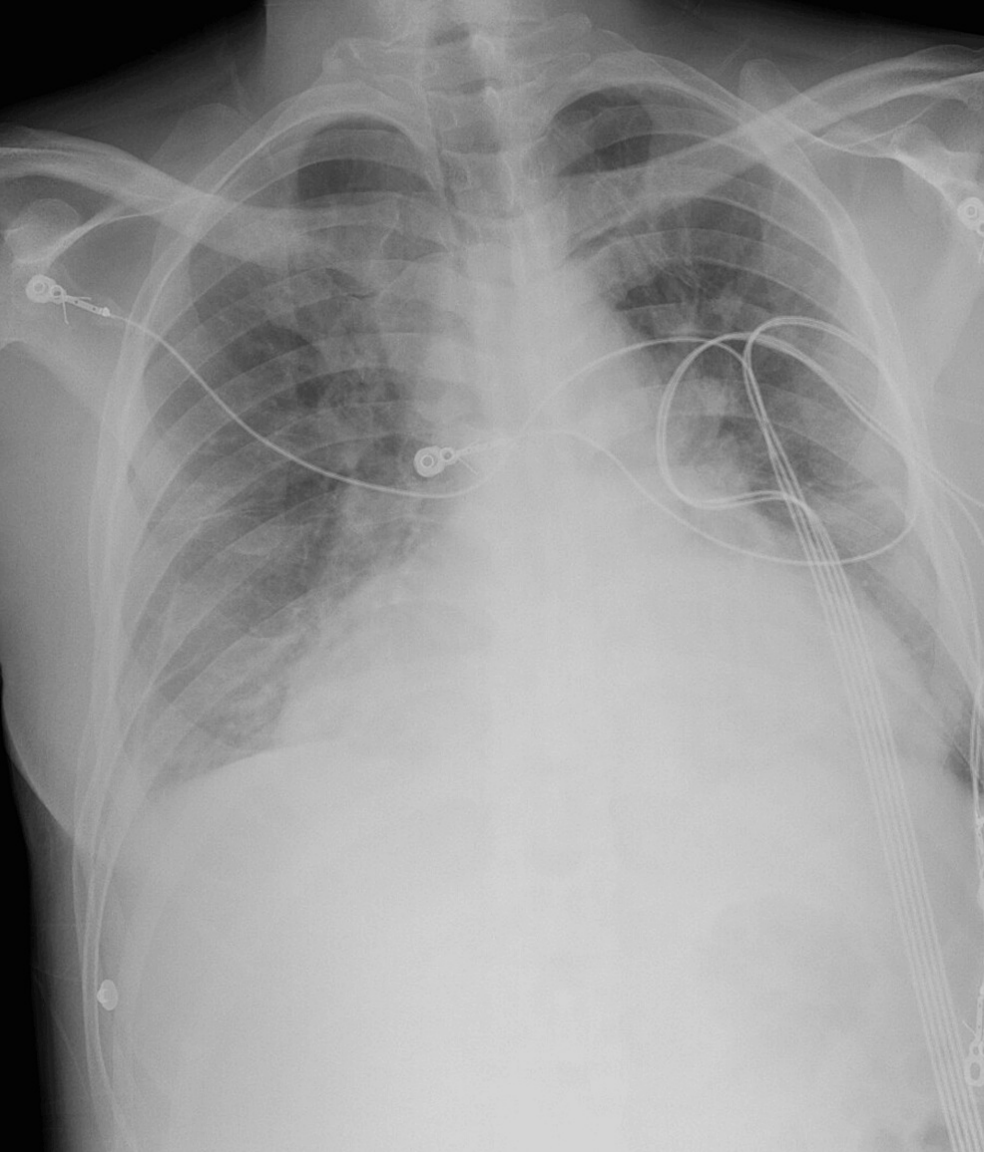
Chest radiograph shows severe enlargement of the cardiac silhouette and pulmonary edema.

**Figure 2:**
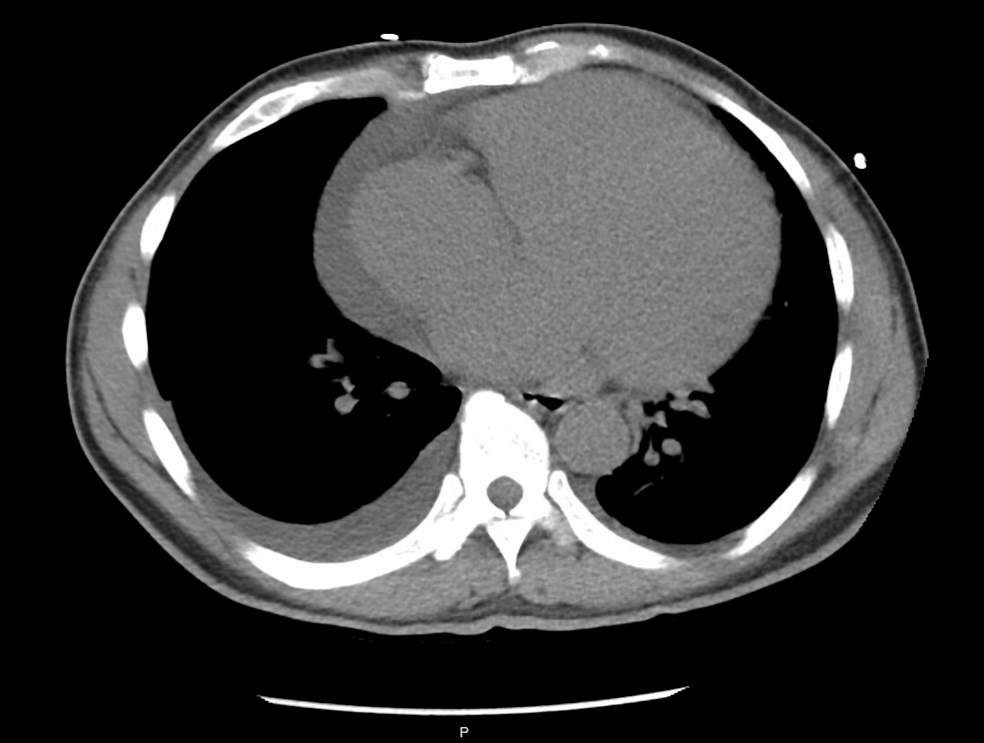
CT angiogram of the chest reveals severe cardiomegaly, a moderate pericardial  effusion, and bilateral pleural effusions.

Initially, the patient had a temperature of 97.3°F, blood pressure (BP) of 232/177 mmHg, heart rate (HR) of 122 beats/minute, respiratory rate (RR) of 18 breaths/minute, oxygen saturation (SpO_2_) of 100% on room air and body mass index (BMI) of 25.8 kg/m^2^. His ECG was abnormal for sinus tachycardia with voltage criteria for LVH. Initial comprehensive metabolic panel (CMP) was notable for sodium 137 mEq/L (reference range 135-145 mEq/L), potassium 3.3 mEq/L (reference range 3.4-5.1 mEq/L), chloride 96 mEq/L (reference range 97-110 mEq/L), bicarbonate 27 mEq/L (reference range 21-32 mEq/L), anion gap 13 mmol/L (reference range 7-9 mmol/L), urea 33 mg/dL (reference range 6-20 mg/dL) and creatinine 2.4 mg/dL (reference range 0.39-0.90 mg/dL). Chest radiograph (Figure 1) followed by a CT angiogram of the chest (Figure 2) were notable for severe cardiomegaly, moderate pericardial effusion, moderate pulmonary edema, and bilateral pleural effusions. After initial resuscitation in the ED, his BP decreased to 171/131 mmHg. The was subsequently admitted to the ICU for further workup and management.

**Figure 3:**
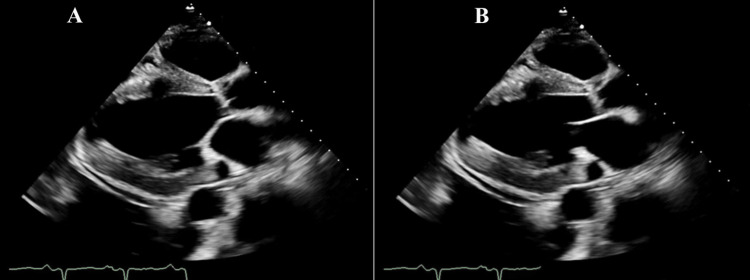
On transthoracic echocardiogram, parasternal long-axis views of the heart in end-systole (A) and end-diastole (B) show dilation and hypertrophy of the left ventricle as well as left atrial dilation. There is a small difference between the diameter of the left ventricle at the end of systole (A) and the end of diastole (B) indicating global left ventricular hypokinesia.

**Figure 4:**
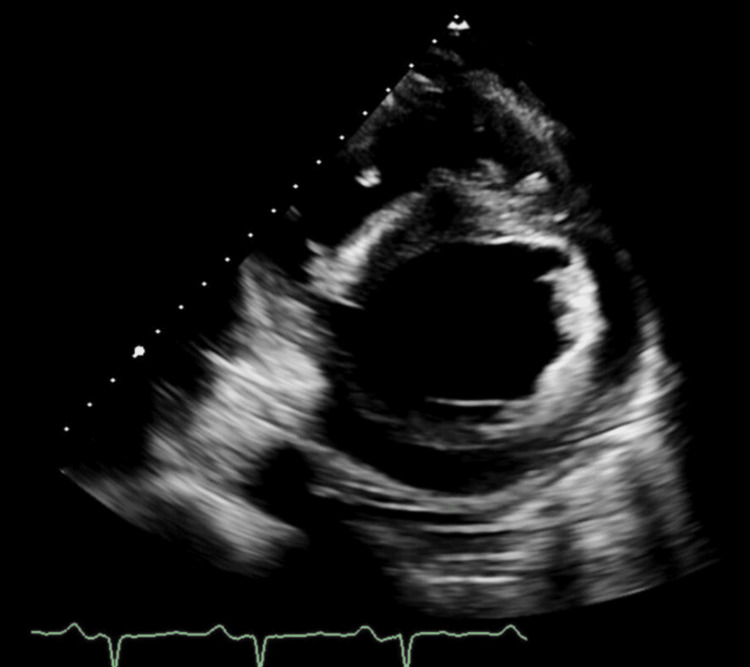
On transthoracic echocardiogram, parasternal short-axis view of the heart in diastole shows concentric left ventricular hypertrophy.

On admission, physical exam was notable for the presence of jugular venous distension (JVD) to the angle of the mandible. On cardiac exam, there was regular heart rate and rhythm with a normal S1 and S2. An S3 was appreciated. On auscultation of his lungs, diffuse rales of bilateral lung fields were heard. His lower extremities were with 3+ pitting edema bilaterally. Transthoracic echocardiogram (Figures 3, 4) was remarkable for the following: mild concentric left ventricular hypertrophy, severely decreased systolic function with an LV ejection fraction (LVEF) of approximately 10-15%, severe global hypokinesis, grade III (severe) diastolic dysfunction, right ventricular cavity dilation, moderate-to-severe reduction in systolic function, mild-to-moderate left atrial enlargement, mildly dilated right atrial cavity, aortic sclerosis, trace aortic regurgitation, thickening of the mitral valve leaflets with severely reduced excursion, mild mitral regurgitation, mild dilation of the tricuspid valve annulus, mild-to-moderate tricuspid valve regurgitation, severe pulmonary HTN with PAs of approximately 70 mmHg, mild pulmonary regurgitation with a mean pulmonary artery pressure of approximately 45 mmHg, mild-to-moderate pericardial effusion adjacent to the right atrium and the right ventricle as well as a dilated inferior vena cava with abnormal respiratory variation. Bilateral renal ultrasound showed diffuse renal parenchymal echogenicity in the setting of medical renal disease and no obstruction. Renal duplex sonography was negative for renal artery stenosis.

The patient was started on furosemide 60 mg IV every 12 hours. Despite the addition of hydralazine 50 mg per os or by mouth (PO) every eight hours followed by isosorbide dinitrate (Isordil) 20 mg PO every eight hours, there was no significant change in his BP. After 24-48 hours, despite increasing hydralazine therapy to hydralazine 100 mg PO every eight hours as well as adding carvedilol 6.25 mg PO twice daily (BID) and Entresto 24-26 mg PO BID, the patient continued to have markedly elevated BP readings with systolic BP of 171-251 mmHg and diastolic BP of 129-173 mmHg. Additionally, despite appropriate potassium and magnesium replacement, the patient continued to be hypokalemic (potassium 2.9-3.4 mEq/L). Further testing yielded a PAC of 27.3 ng/dl and PRA of 0.7 ng/ml/hr. The aldosterone-renin ratio (ARR) was calculated to be 39.0, suggesting PA.

**Figure 5:**
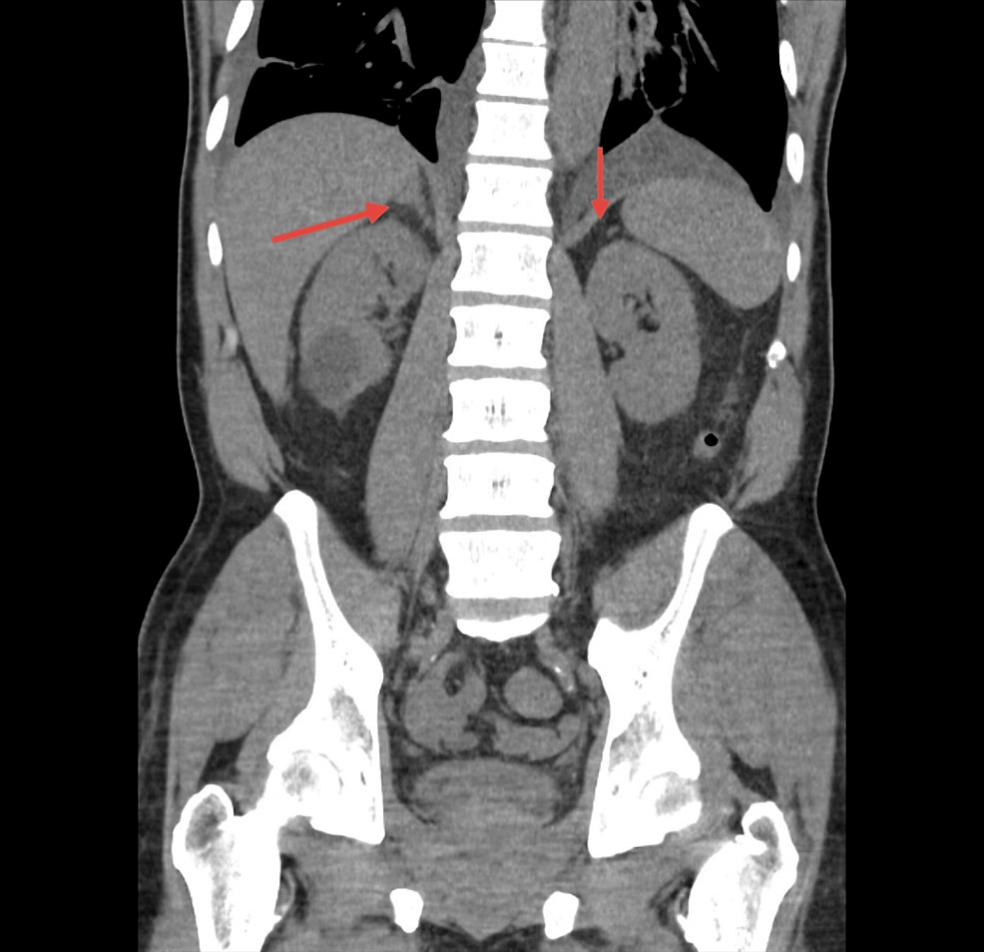
Non-contrast CT of the abdomen and pelvis does not portray any adrenal nodules.

Non-contrast CT of the abdomen and pelvis did not show any adrenal nodules (Figure 5). Further adrenal-specific imaging and adrenal-venous sampling were determined to be of low utility as the patient was deemed to be a poor candidate for any surgical intervention. The patient was started on an MRA, specifically spironolactone 25 mg PO BID. After the initial dose of spironolactone 25 mg PO, the patient's BP decreased from 181/121 mmHg to 139/86 mmHg. In the next 24-48 hours, hydralazine and Isordil were discontinued, and guideline-directed medical therapy (GDMT) for HF with reduced ejection fraction (HFrEF) was optimized. On discharge, the patient’s BP was 119/67 mmHg. He was discharged with spironolactone 25 mg BID, sacubitril/valsartan (Entresto) 24-26 mg BID, carvedilol 12.5 mg BID, and torsemide 25 mg as needed for fluid overload. ​​​​​

The etiology of the patient’s dilated cardiomyopathy (DCM) was thought to be secondary to hypertensive heart disease, alcoholic cardiomyopathy, and/or viral myocarditis. In the setting of the patient's renal dysfunction and otherwise low suspicion for an ischemic DCM, an ischemic evaluation was not conducted during the patient's inpatient hospital admission. The patient was advised to follow up with cardiology after discharge in the outpatient setting for diagnostic right and left heart catheterizations with CTA coronary or invasive angiography in order to definitively assess for cardiac ischemia as well as for a cardiac MRI in order to further explore the possibility of a myocarditis-induced DCM.

## Discussion

Normally, excess circulating aldosterone suppresses the release of renin from the juxtaglomerular cells of the nephron [8]. This decreases the production of angiotensin-II, which in turn decreases the secretion of aldosterone from the adrenal glands. In PA, there is a renin-angiotensin-aldosterone system (RAAS)-independent secretion of aldosterone from the adrenal gland(s). Aldosterone inserts epithelial sodium channels (ENaC) on the distal collecting tubule (DCT) and the inner medullary collecting duct of the nephron such that Na+ (which indicates volume/preload) is reabsorbed, increasing extracellular volume and BP. Aldosterone also activates Na-K-ATPase channels in the basal membrane, which results in a net loss of potassium into the tubular lumen, and elevates carbonic anhydrase, resulting in the reabsorption of bicarbonate (HCO_3_-) and excretion of hydron (H+) [8]. Because MRAs inhibit aldosterone by competitively binding to receptors at the aldosterone-dependent Na/K exchange pump in the DCT, they are the mainstay pharmacotherapy for the treatment of PA.

The Randomized Aldactone Evaluation Study (RALES) found that in patients with HFrEF (i.e. LVEF <35%) and New York Heart Association (NYHA) class III-IV symptoms, spironolactone (in comparison to placebo) significantly reduced CV hospitalizations and CV-related mortality and was also associated with an improvement in NYHA class [8,9]. The Eplerenone in Mild Patients Hospitalization and Survival Study in HF (EMPHASIS-HF) trial determined that compared to placebo, eplerenone reduced the risk of hospitalization and death in patients with moderate systolic dysfunction and NYHA class II symptoms [10]. Subsequently, a meta-analysis found that MRAs provide a relative risk reduction of 25% in all-cause mortality and 23% in hospitalizations for HF [11]. 

Presently, the American Heart Association (AHA)/American College of Cardiology Foundation (ACCF) HF Guidelines recommend MRA pharmacotherapy for patients with HFrEF in NYHA class III-IV as well as for patients in NYHA class II with a prior CV-related hospitalization and/or an elevated B-type natriuretic peptide [11]. A one-to-three-month delay in initiation of MRA pharmacotherapy after discharge from initial hospitalization for decompensated HF has been linked to a significantly increased mortality compared to initiation of MRA pharmacotherapy at discharge [12]. The beneficial effects of MRAs on survival in HF may at least be in part mediated by restoring cardiac norepinephrine (NE) homeostasis [13]. Cardiac NE homeostasis is largely dependent on the proper functioning of the NE transporter (NET), which removes 90% of NE after it is released into the synaptic cleft via neuronal reuptake. In HF, decreased NET function increases the concentration of NE at postsynaptic adrenoceptors, depleting cardiac NE stores and hence, down-regulating cardiac beta-adrenoceptors. Resultant cardiac myocyte apoptosis causes cardiac remodeling, cardiac dysfunction, and arrhythmias in HF. A high-salt diet significantly diminishes NET-mediated NE reuptake. An elevated plasma level of aldosterone enhances cardiac sympathetic activity, amplifying this negative effect. Thus, in comparison to patients with uncontrolled essential HTN, patients with unmanaged PA have a greater degree of LV remodeling [13].

## Conclusions

By starting our patient on spironolactone, we corrected his hypertensive emergency in the acute setting. The patient continued spironolactone for the medical management of PA and for the treatment of HFrEF with GDMT. Not only are MRAs the mainstay therapy for the treatment of PA, they have also been shown to reduce mortality in patients with HFrEF. Initiation of MRA pharmacotherapy upon discharge from hospitalization for acutely decompensated HF has been shown to significantly reduce mortality in patients with HFrEF.

## References

[1] Funder JW, Carey RM, Fardella C (2008). Case detection, diagnosis, and treatment of patients with primary aldosteronism: an endocrine society clinical practice guideline. J Clin Endocrinol Metab.

[2] Mulatero P, Stowasser M, Loh KC (2004). Increased diagnosis of primary aldosteronism, including surgically correctable forms, in centers from five continents. J Clin Endocrinol Metab.

[3] Funder JW, Carey RM, Mantero F (2016). The management of primary aldosteronism: case detection, diagnosis, and treatment: an Endocrine Society clinical practice guideline. J Clin Endocrinol Metab.

[4] Quencer KB (2021). Adrenal vein sampling: technique and protocol, a systematic review. CVIR Endovasc.

[5] Young WF Jr (2019). Diagnosis and treatment of primary aldosteronism: practical clinical perspectives. J Intern Med.

[6] Mahmud A, Feely J (2005). Aldosterone-to-renin ratio, arterial stiffness, and the response to aldosterone antagonism in essential hypertension. Am J Hypertens.

[7] Tsai CH, Pan CT, Chang YY, Chen ZW, Wu VC, Hung CS, Lin YH (2021). Left ventricular remodeling and dysfunction in primary aldosteronism. J Hum Hypertens.

[8] McManus F, Connell J (2009). Safety and efficacy of eplerenone in the management of essential hypertension. Clin Med Ther.

[9] Pitt B, Zannad F, Remme WJ (1999). The effect of spironolactone on morbidity and mortality in patients with severe heart failure. Randomized Aldactone Evaluation Study Investigators. N Engl J Med.

[10] Zannad F, McMurray JJ, Krum H (2011). Eplerenone in patients with systolic heart failure and mild symptoms. N Engl J Med.

[11] Dixit NM, Shivani S, Boback Z (2021). Optimizing guideline-directed medical therapies for heart failure with reduced ejection fraction during hospitalization. US Cardiology Review.

[12] Rossi R, Crupi N, Coppi F, Monopoli D, Sgura F (2015). Importance of the time of initiation of mineralocorticoid receptor antagonists on risk of mortality in patients with heart failure. J Renin Angiotensin Aldosterone Syst.

[13] Kreusser MM, Lehmann LH, Haass M, Buss SJ, Katus HA, Lossnitzer D (2017). Depletion of cardiac catecholamine stores impairs cardiac norepinephrine re-uptake by downregulation of the norepinephrine transporter. PLoS One.

